# Immunology of Wound Healing

**DOI:** 10.1007/s13671-018-0234-9

**Published:** 2018-09-28

**Authors:** Samantha Ellis, Elaine J. Lin, Danielle Tartar

**Affiliations:** Department of Dermatology, University of California, Davis, 3301 C Street, Ste. 1300, Sacramento, CA 95816 USA

**Keywords:** Wound healing, Chronic wound, Neutrophil, Macrophage, Anti-inflammatory macrophage, Re-epithelialization

## Abstract

**Purpose of Review:**

Chronic wounds are a tremendous burden on the healthcare system and lead to significant patient morbidity and mortality. Normal cutaneous wound healing occurs through an intricate and delicate interplay between the immune system, keratinocytes, and dermal cells. Each cell type contributes signals that drive the normal phases of wound healing: hemostasis, inflammation, proliferation, and remodeling. This paper reviews how various immunological cell types and signaling molecules influence the way wounds develop, persist, and heal.

**Recent Findings:**

Concurrent with the achievement of hemostasis, neutrophils are the first cells to migrate to the wound bed, brought in by pro-inflammatory signals including IL-8. Their apoptosis and engulfment by macrophages (efferocytosis) provides a key signal to the local immune milieu, including macrophages, to transition to an anti-inflammatory, pro-repair state, where angiogenesis occurs and granulation tissue is laid down. Myofibroblasts, activated through contractile forces and signaling molecules, then drive remodeling, where granulation tissue becomes scar. Unchecked inflammation at this stage can result in abnormal scar formation.

**Summary:**

Although the derangement of immune signals at any stage can result in impaired wound healing, recent research has shown that the key transition point lies between the inflammatory and the proliferative phases. This review summarizes the events that facilitate this transition and discusses how this process can be disrupted, leading to chronic, non-healing wounds.

## Introduction

In the USA, chronic wounds afflict approximately 6.5 million individuals, leading the healthcare system to spend over 25 billion dollars, annually, on their treatment [1]. Non-healing wounds are more than just a cost burden, as they have been shown to cause loss of mobility and ability to perform daily tasks, loss of participation in the workforce, and poor quality of life [2, 3]. The effect of non-healing wounds on mortality has even been demonstrated to be comparable to cancer [4]. As the population continues to age, and rates of obesity, diabetes, and cardiovascular disease rise, the number of chronic wounds worldwide is expected to rise as well [5•].

**Fig. 1:**
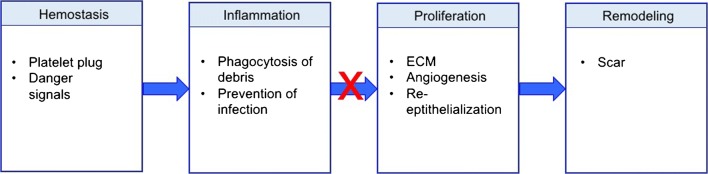
Legend: traditional model of wound healing. Wound healing normally progresses through the hemostasis/inflammatory phase, the proliferative phase, and the remodeling phase. Hemostasis is achieved with production of a fibrin clot. Danger signals are released from platelets and damaged cells, which leads to infiltration and activation of pro-inflammatory cells such as neutrophils and inflammatory-type macrophages. There is an important transition from the inflammatory to the proliferative phase (days 2–5). In chronic wounds, this transition often fails to occur. In the proliferative phase, extracellular matrix (ECM) is laid down to form granulation tissue, and angiogenesis and re-epithelialization occur. Over the next year, the granulation tissue is remodeled into a scar

Given the tremendous strain that chronic wounds place on the healthcare system, considerable efforts are underway to investigate the basic science of wound healing and to understand the conditions that lead to chronic wounds. In particular, the immune system has been found to play a substantial role due to its impact on several repair mechanisms [6, 7]. Though the process of wound healing is markedly complex and dependent on the delicate interplay of numerous factors, normal wound healing can generally be broken down into four over-lapping but distinct steps (Fig. 1). These steps include hemostasis (minutes to hours after injury), inflammation (days 1–3), proliferation and repair (days 4–21), and lastly, wound remodeling (days 21–365) [8, 9]. Dysregulation of any of these events can result in delayed wound healing and the potential to form chronic ulcers and/or excessive scarring [10••]. This review summarizes the events taking place in each stage of wound healing, with a focus on immune pathways and how they are disturbed in chronic wounds. New research has illustrated that chronic wounds fail to shift from the inflammatory to the proliferative phase of wound healing, so much of this review will focus on the events that drive this transition.

## Four Stages of Wound Healing

### Hemostasis

The initial events following injury are designed to achieve hemostasis within the first minutes to hours of injury based on a series of serine protease events designed to prevent blood loss [11]. In this cascade, a series of biologically inert zymogens (enzyme precursors) are activated into fully functional, catalytically active, serine proteases that result in the platelet activation and formation of a fibrin clot. Platelet activation not only results in hemostasis, but also in the release of growth factors such as platelet-derived growth factor (PDGF) as well as immune mediators that are responsible for activation of the immune system and transition to the inflammatory phase of wound healing.

The hemostasis phase begins when tissue damage allows blood to leak into the exposed wound site, triggering the extrinsic clotting cascade and releasing mediators that cause localized vasoconstriction, such as serotonin [9]. Platelets subsequently aggregate and activate on subendothelial collagen, leading to formation of a hemostatic plug through their release of cytokines and growth factors [12]. Not only does this mitigate hemorrhage, but also serves as a preliminary matrix for cell migration by releasing scaffold proteins such as fibronectin, vitronectin, and thrombospondins, allowing for the migration of keratinocytes, immune cells, and fibroblasts [9, 13]. Platelet degranulation also leads to the release of inflammatory mediators such as interleukin (IL)-8, or CXCL8 (a potent neutrophil chemoattractant), in addition to IL-1α, IL-1β, IL-6, and tumor necrosis factor (TNF)-α, and activates the complement cascade [9, 14]. After hemostasis is achieved, histamine released by the activated complement cascade causes capillary dilation and leakage, accelerating migration of inflammatory cells into the wound bed and full transition to the inflammatory phase of wound healing [15].

### Inflammation

The inflammatory phase overlaps considerably with initial hemostasis, occurring during the first 72 h after tissue injury [16]. This phase is principally represented by a complex series of molecular signals that ultimately facilitates neutrophil and monocyte infiltration of the wound bed in order to prevent unnecessary tissue damage and eliminate pathogenic organisms and foreign debris [6, 16].

Inflammatory cell recruitment into the wound site occurs secondary to local stimuli. In an acute wound, injured host cells die and release cellular contents that serve as danger signals (e.g., uric acid, DNA, RNA, extracellular matrix components). These products are collectively referred to as damage-associated molecular patterns (DAMPs) [17, 18]. When a wound is contaminated by a pathogen, pathogen-associated molecular patterns (PAMPs) are also released into the wound milieu [19]. Pattern recognition receptors (e.g., toll-like receptors) on local, tissue-resident cells recognize these danger signals, which leads to local cell activation. Subsequently, these cells express numerous genes that code for important chemical mediators that will propagate the inflammatory response [20, 21].

#### Neutrophil Activation and Amplification

Neutrophils represent the most abundant inflammatory cells to infiltrate a new wound and function mainly to remove debris and prevent infection [22, 23]. Their influx is mediated by a number of chemical signals, including IL-8, or CXCL8, as mentioned above, and neutrophils have over 30 different receptors that mitigate their migration and activation response [24]. It is clear that neutrophils do function in debris removal early in wound healing, but their persistence, as will be discussed in detail below, has been associated with delayed wound healing and chronic wounds. Moreover, mouse models of wound healing have shown that in non-aged, non-impaired models, neutrophil depletion does not negatively affect wound healing as profoundly as macrophage deletion [25–27]. In impaired models of wound healing, such as diabetes, where infection risk is higher, neutrophils are clearly required [28].

DAMPs, released from necrotic cells, are thought to be the first signals to recruit neutrophils to the wound bed [29]. These danger signal molecules can activate neutrophils directly by binding various neutrophil surface receptors, in addition to signaling tissue-resident cells to produce neutrophil chemoattractants [23, 30].

One of the most well-described chemoattractants produced by tissue-resident macrophages and fibroblasts is CXCL8 (IL-8) [31]. CXCL8 binds and stimulates neutrophil surface receptors CXCR1 and CXCR2, leading to avid recruitment of neutrophils to the site of tissue injury [32, 33]. Interestingly, once neutrophils migrate into the wound they are also able to secrete CXCL8, creating a pro-inflammatory feedback loop [34]. Endothelial permeability is also increased by CXCL8, further encouraging inflammatory cell influx into the wound site [35]. Other CXCL8 family chemokines, such as CXCL1, CXCL2, CXCL3, CXCL5, CXCL6, and CXCL7, have also been shown to play a role in neutrophil chemotaxis [18, 34, 36]. By binding glycosaminoglycans on tissue cell walls and in the extracellular matrix, these chemokines, including CXCL8, create a signaling gradient to allow for clear directional migration of neutrophils towards the injury [18, 34, 37, 38]. Additional DAMP-induced cellular byproducts, such as hydrogen peroxide (H_2_O_2_) and leukotriene B_4_ (LTB_4_), also form gradients to drive focused migration of neutrophils [23].

#### Neutrophils in Wound Healing

Although neutrophils are not considered an essential cell type in non-impaired wound healing, they do carry out a variety of functions that support the process [27, 39]. First and foremost, neutrophils defend against wound infection by phagocytosing pathogens then killing them through release of reactive oxygen species, proteases, or antimicrobial proteins [28]. With degranulation, antimicrobial proteins can also be released into the surrounding milieu to destroy extracellular organisms [40]. More recent evidence indicates that neutrophils can also eliminate organisms residing in the extracellular environment through the deployment of neutrophil extracellular traps (NETs). NETs are web-like structures comprised of strands of decondensed chromatin bound to neutrophil-produced bactericidal proteins. They work by either directly killing microorganisms or via immobilizing pathogens to facilitate phagocytosis [41, 42].

In addition to clearing pathogens, neutrophils also regulate inflammation and generate growth factors and cytokines to induce wound healing. In the wound environment, neutrophils have exhibited the ability to upregulate gene expression of chemokines that are key recruiters of macrophages, T cells, and additional neutrophils, such as TNF-α, IL-1β, IL-6, CXCL8, CXCL2, and monocyte chemoattractant protein-1 (MCP-1) [16, 34, 43]. Neutrophils also show increased expression of cytokines that promote angiogenesis [e.g., vascular endothelial growth factor (VEGF), CXCL3, and MCP-1], proliferation of fibroblasts and keratinocytes (IL-8, IL-1 β, and MCP-1), adhesion of keratinocytes to the dermal layer (laminin 5 β-3), and tissue remodeling [urokinase-type plasminogen activator (uPA)] [34, 43–45].

#### Neutrophils in Chronic Wounds

While neutrophils do play an important role in propagating the inflammatory response in the early stages of wound healing, they also serve as a signal to inactivate the inflammatory phase [46]. In physiologic wound repair, neutrophils undergo apoptosis after carrying out their various functions at the site of injury. Local macrophage uptake of apoptotic neutrophils then triggers a transition out of inflammatory phase [47–49]. More recent studies also indicate that some neutrophils may actually undergo reverse migration, away from the site of injury and back into circulation. This is called reverse transendothelial migration (rTEM), and serves two potential functions: a mechanism to resolve local inflammation and/or a mechanism to redistribute activated neutrophils to other locations in the body, leading to inflammation at other sites [23].

Although the recruitment of neutrophils is crucial in host protection, the associated robust inflammatory response may also be detrimental to proper wound healing [28, 50, 51]. Many studies suggest that the prolonged presence of neutrophils and their associated inflammatory mediators in the wound milieu contributes to the formation and persistence of chronic wounds. For example, neutrophil-derived proteases, such as elastase and matrix metalloproteinases (MMPs), can degrade healthy extracellular matrix (ECM), and increased levels of these proteases have been repeatedly detected in chronic wounds [52–56]. Neutrophils can also generate deleterious levels of reactive oxygen species in chronic wounds, damaging cell membranes and causing additional destruction of the ECM. This destruction encourages additional production of inflammatory mediators (e.g., IL-1, TNF-α) and proteolytic enzymes (e.g., MMPs), propagating a cycle of inflammation amplification [28, 57]. Thus, it comes as no surprise that chronic wounds demonstrate significantly increased levels of the potent neutrophil chemoattractant, CXCL8 [58].

NETs have also been detected in excess in diabetic foot wounds and have been shown to delay healing, and inhibition of NETosis and NET function in mouse models of delayed wound healing improves outcomes [59•]. In sum, the sustained and inappropriate presence of neutrophils at the site of injury is a major contributing factor in non-healing wounds.

#### Macrophage Activation and Inflammation Amplification

**Fig. 2:**
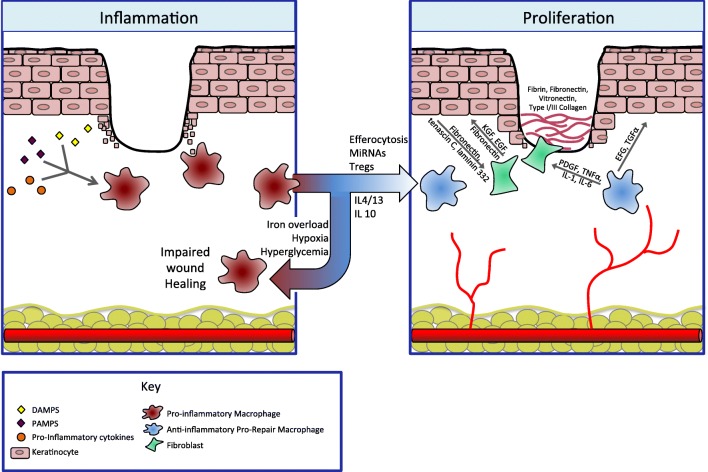
Legend: the transition from pro-inflammatory macrophages to anti-inflammatory macrophages is a key regulatory step, allowing the immune system to promote both ECM formation and re-epithelialization. During the inflammatory phase, pro-inflammatory macrophages dominate. They are activated by danger signals such as pathogen-associated molecular patterns (PAMPs) and danger-associated molecular patterns (DAMPs) as well as pro-inflammatory cytokines. This phenotype is responsible for clearance of debris and prevention of infection. Persistence of inflammation results in a non-healing wound. Normally, macrophages transition to an anti-inflammatory phenotype in response to signals such as neutrophil apoptosis and engulfment (efferocytosis) as well as other local immune signals. This transition is inhibited in the setting of iron overload, hypoxia, and hyperglycemia. These pro-healing, anti-inflammatory macrophages are responsible for resolution of tissue inflammation and contribute to angiogenesis and tissue repair. During the proliferative phase, new blood vessels and granulation tissue are laid down and keratinocytes re-epithelialize. Pro-repair macrophages send signals to both fibroblasts and keratinocytes themselves. To keratinocytes, they release epidermal growth factor (EGF) and transforming growth factor-α (TGF-α), which drive keratinocyte proliferation and migration. Through platelet-derived growth factor (PDGF), TNF-α, IL-1, and IL-6, pro-repair macrophages signal fibroblasts to lay down granulation tissue, comprised of fibrin, fibronectin, as well as collagen. In turn, fibroblasts further stimulate keratinocyte proliferation and migration through keratinocyte growth factor (KGF), EGF, and fibronectin. Keratinocytes themselves also activate fibroblasts in a feedback loop through the production of fibronectin, tenascin C, and laminin 332

Macrophages play a critical role in wound healing, and their roles in angiogenesis, fibroplasia, cell proliferation, and transition out of the inflammatory phase is clear (Fig. 2) [25–27, 60]. At baseline, macrophages are phagocytic monocyte-derived cells that constitutively scavenge and remove dead cells, necrotic tissue, and toxic metabolites from the tissues [61]. However, after injury, these homeostatic functions are amplified by a variety of stimuli in order to facilitate tissue repair.

With cutaneous injury, local, skin-resident macrophages become activated via danger signals and other injury-by-product molecules (e.g., H_2_O_2_), while monocytes in circulation exit blood vessels and enter the wound site. It was previously thought that neutrophils were the only inflammatory cells infiltrating a wound immediately after injury; however, a recent study demonstrated that a surge of monocytes enter the wound bed simultaneously, traveling through sites of vascular leakage [62, 63].

DAMPs and PAMPs (released from necrotic tissue and pathogens, respectively), as well as interferon-γ [(IFN-γ), released from natural killer cells] polarize macrophages into a pro-inflammatory phenotype [64]. These inflammatory macrophages are often referred to as classically activated macrophages, or the M1 phenotype [65]. This phenotype secretes pro-inflammatory cytokines, such as IL-1β, IL-6, IL-12, IL-23, and TNF-α, as well as chemokines that induce increased natural killer cell, macrophage, and helper T cell responses [65–67]. These inflammatory amplification molecules are essential, because prior to injury, the skin has relatively few resident macrophages, and the majority of wound-related macrophages are derived from intravascular monocytes [68, 69]. In addition to signaling recruitment of leukocytes to the wound bed, these M1 macrophages demonstrate an increased capacity to destroy and phagocytose microbes and cellular debris to keep the site of injury clean [70–72].

#### Anti-inflammatory Macrophages

Macrophages in the wound site are responsible for phagocytosis of apoptotic neutrophils, a process known as efferocytosis [73••]. This action itself induces macrophages to transition from a pro-inflammatory phenotype, to an anti-inflammatory phenotype, often referred to as M2 or “alternatively activated” macrophages [47]. Rather than be considered distinct M1 and M2 cell types, it is better to characterize these macrophages as existing on a spectrum of activation between pro-inflammatory and anti-inflammatory [74]. Other mediators that induce this transition include glucocorticoids, IL-10, prostaglandins, the IL-4/IL-13 pathway, and engagement of specific toll-like receptors (TLRs) [26, 60, 75]. Interestingly, while IL-4/13 are the primary signals to induce this phenotype in vitro, recent studies have shown that they are not required in vivo [26, 76]. More recent work has demonstrated roles for regulatory T cells (Tregs), adenosine signaling, and microRNAs (miRNAs) [10••, 77, 78]. Anti-inflammatory macrophages represent a more heterogeneous cell population, and are comprised of all macrophages that do not have the pro-inflammatory phenotype [79]. These cells tamper down inflammation and stimulate tissue repair by generating anti-inflammatory molecules such as IL-1 receptor antagonist and IL-10, as well as growth factors that promote ECM synthesis, angiogenesis, and fibroblast proliferation, such as transforming growth factor-β (TGF-β) and VEGF [80]. Transitioning from a pro-inflammatory macrophage-dominant wound to an anti-inflammatory macrophage-dominant milieu is essential in resolving inflammation and preparing the wound for effective repair [72].

#### Macrophages in Chronic Wounds

When the transition from a pro- to an anti-inflammatory macrophage phenotype is impaired, wound healing stalls in the inflammatory phase and a chronic wound results [10••]. Certain signals are known to prolong the presence of pro-inflammatory macrophages, including iron overload, which is commonly seen in the skin in the setting of venous stasis [81, 82].

In fact, in a mouse model of iron overload, local wound macrophages persisted in a pro-inflammatory state with excess production of inducible nitric oxide synthase (iNOS), IL-12, and TNF-α, in addition to free radicals, leading to impaired wound healing [81]. In addition to increased pro-inflammatory molecule expression, iron-overloaded macrophages showed decreased levels of anti-inflammatory markers such as IL-4, IL-10, and CD206 [81]. Macrophages from patients with chronic, non-healing, wounds have been shown to have higher levels of iron, based on Prussian blue staining [82, 83].

Additional signals, such as hyperglycemia in the setting of diabetes, hypoxia in the setting of arterial or venous insufficiency, or secondary infection likely also contribute to the persistence of pro-inflammatory macrophages.

### Proliferation

As inflammation resolves, the proliferation phase begins. This involves re-establishing vascular channels, generating granulation tissue, and re-epithelializing the wound surface. In physiologic wound repair, keratinocytes from the wound edge begin migrating centrally within hours of tissue injury, and epithelial stem cells from the basal layer of the epidermis and hair follicle root sheath begin proliferating 2–3 days after tissue injury [84]. New blood vessel formation and re-epithelialization occurs secondary to multiple chemical and physical signals, some of which come from immune cells, including anti-inflammatory, pro-repair macrophages.

Restoring the vascular network is an important part of the proliferative phase. New blood vessel formation, referred to as angiogenesis, occurs in a two-step process: vessel sprouting, followed by vessel anastomosis [85]. Not only do anti-inflammatory, pro-repair macrophages produce VEGF, which promotes vessel sprouting, they also express two transmembrane proteins that have been shown to promote vascular anastomosis [80, 85, 86]. This function of macrophages is vital to effective wound healing, and, in macrophage-deficient models, angiogenesis is impaired [85].

The formation of granulation tissue, which is comprised primarily of type III collagen, fibroblasts, and new blood vessels, occurs contemporaneously with angiogenesis. Fibroblasts are the main cell involved in granulation tissue formation, and several macrophage-derived molecules, such as platelet-derived growth factor β-bb (PDGF-bb), TNF-α, IL-1, and IL-6, can induce pro-re-epithelialization molecules in fibroblasts (Fig. 2) [87, 88]. Wounds without IL-6 lack an appropriate inflammatory response and demonstrate stunted angiogenesis, collagen accumulation, and re-epithelialization [89]. Fibroblasts are also exceptionally influenced by TGF-β, a molecule predominantly produced by wound-associated, pro-repair macrophages [90, 91].

Keratinocyte re-epithelialization is influenced by both fibroblasts in the granulation tissue and pro-repair macrophages. Re-epithelialization is initiated by epidermal growth factor (EGF), keratinocyte growth factor (KGF), and transforming growth factor-α (TGF-α), which are produced by platelets, keratinocytes, and activated pro-repair, anti-inflammatory macrophages [92]. Keratinocytes themselves further activate fibroblasts in a feedback loop through the production of fibronectin, tenascin C, and laminin 332 (Fig. 2) [93].

### Repair/Remodeling

#### Wound Remodeling and Contraction

Remodeling starts several weeks after wounding, and continues for up to 1 year. It marks the transition from granulation tissue to scar, which involves the slowing of angiogenesis and replacing type III collagen in granulation tissue with stronger type I collagen. It should be noted that fully mature scars return to only 80% of their initial tensile strength [10••]. This remodeling phase is largely driven by myofibroblasts, which develop from fibroblasts in response to both mechanical tension and TGF-β signaling and are responsible for contraction of the wound [94].

Myofibroblasts express smooth muscle actin (SMA), which is responsible for generating the contractile force attributed to this cell type [94, 95]. In addition to contraction of wound beds and generation of collagen, myofibroblasts contribute to remodeling by release of MMPs that degrade collagen laid down during granulation tissue formation [96, 97]. Conventional dogma states that myofibroblasts are terminally differentiated and undergo apoptosis following wound remodeling. However, exciting new research indicates that these wound bed myofibroblasts may further differentiate into fat cells, replenishing subcutaneous adipose tissue. This process is dependent upon neogenic hair follicles, which lead to bone morphogenic protein (BMP) signaling and activation of adipocyte transcription factors [98••].

#### Hypertrophic and Keloid Scar Formation

When scar formation is excessive, the scar itself can lead to pruritus, pain, or a disfiguring appearance. While excess inflammation may lead to either keloid or hypertrophic scar formation, there are several key clinical differences. Hypertrophic scars arise within 1–2 months of injury, and tend to arise in areas of high tension. Keloid scars can occur at any point post-injury, do not tend to occur in areas of high tension, and may grow beyond the borders of the initial scar [99].

Normally, myofibroblasts carefully coordinate the breakdown of granulation tissue and replacement with long-lasting type I collagen [100]. Recent evidence indicates that myofibroblast-induced fibrosis can be over-activated, not only in the setting of signaling through TGF-β, but also in the setting of Th2-derived cytokines IL-4 and IL-13; Th1 cytokines, such as IFN-γ, attenuate excessive scar formation [100, 101]. In fact, clinical trials are currently underway to explore anti-IL-4/IL-13 therapies in pulmonary fibrosis [101]. A role for these therapies in cutaneous fibrosis remains to be explored, though in vitro studies have shown that blocking signaling of the related cytokine IL-10 in fibroblasts may decrease keloid scar formation [102].

## Conclusions

Cutaneous wound healing occurs through an intricate and delicate interplay between the immune system, keratinocytes, and dermal cells such as platelets, fibroblasts, and myofibroblasts. Each cell type contributes proteins and molecular signals that transition the cycle through the normal phases of wound healing, including the hemostasis, inflammatory, proliferative, and remodeling phases. Alteration of normal signals at any stage can result in impaired wound healing, with non-healing wounds or excessive scar formation, at great cost to both patients and the healthcare system.

Recent research has shown that the key transition point in wound healing lies between the inflammatory and the proliferative phases. Numerous signals are responsible for the transition, most notably the apoptosis and phagocytosis of wound-bed resident neutrophils (efferocytosis). If wounds fail to transition from the inflammatory phase, pro-inflammatory macrophages persist and non-healing wounds develop. Future treatments may target this transition point, with an emphasis on generating pro-healing, anti-inflammatory macrophages.
